# CLINICAL MANIFESTATIONS OF CHILDREN AND ADOLESCENTS WITH COVID-19: REPORT OF THE FIRST 115 CASES FROM SABARÁ HOSPITAL INFANTIL

**DOI:** 10.1590/1984-0462/2021/39/2020305

**Published:** 2020-11-27

**Authors:** Anna Clara Rabha, Francisco Ivanildo de Oliveira, Thales Araújo de Oliveira, Regina Grigolli Cesar, Giuliana Fongaro, Roberta Ferreira Mariano, Clarice Neves Camargo, Fátima Rodrigues Fernandes, Gustavo Falbo Wandalsen

**Affiliations:** a Instituto Pensi, Sabará Hospital Infantil, Fundação José Luiz Egydio Setúbal, São Paulo, SP, Brazil.; b Escola Paulista de Medicina, Universidade Federal de São Paulo, São Paulo, SP, Brazil.

**Keywords:** Coronavirus infections, Child, Pneumonia, Infecções por coronavírus, Criança, Pneumonia

## Abstract

**Objective:**

To describe the clinical manifestations and severity of children and adolescents affected by COVID-19 treated at Sabará Hospital Infantil.

**Methods:**

This is a cross-sectional, retrospective, and observational study. All cases of COVID-19 confirmed by RT-qPCR of patients seen at the hospital (emergency room, first-aid room, and ICU) were analyzed. The severity of the cases was classified according to the Chinese Consensus.

**Results:**

Among the 115 children included, a predominance of boys (57%) was verified, and the median age was two years. A total of 22 children were hospitalized, 12 in the ICU. Of the total, 26% had comorbidities with a predominance of asthma (13%). Fever, cough, and nasal discharge were the most frequent symptoms. Respiratory symptoms were reported by 58% of children and gastrointestinal symptoms, by 34%. Three children were asymptomatic, 81 (70%) had upper airway symptoms, 15 (13%) had mild pneumonia, and 16 (14%) had severe pneumonia. Hospitalized children were younger than non-hospitalized children (7 months *vs.* 36 months). In hospitalized patients, a higher frequency of irritability, dyspnea, drowsiness, respiratory distress, low oxygen saturation, and hepatomegaly was observed. Chest radiography was performed in 69 children with 45% of abnormal exams. No child required mechanical ventilation and there were no deaths.

**Conclusions:**

Most of children and adolescents affected by COVID-19 had mild upper airway symptoms. Clinical manifestations of COVID-19 were more severe among younger children who exhibited gastrointestinal and respiratory symptoms more frequently.

## RESUMO

**Objetivo:**

Descrever as manifestações clínicas e a gravidade de crianças e adolescentes acometidos pela COVID-19 atendidos no Sabará Hospital Infantil.

**Métodos:**

Trata-se de estudo transversal, retrospectivo e observacional. Foram analisados os atendimentos (pronto-socorro, enfermaria e Unidade de Terapia Intensiva - UTI) que apresentavam diagnóstico de COVID-19 confirmado por RT-qPCR. A gravidade dos casos foi classificada de acordo com o Consenso Chinês.

**Resultados:**

Entre as 115 crianças incluídas, houve predominância do sexo masculino (57%) e a mediana de idade foi de 2 anos. Vinte e duas crianças foram hospitalizadas, sendo 12 em UTI. Do total, 26% apresentava comorbidades com predomínio de asma (13%). Febre, tosse e coriza foram os sintomas mais frequentes. Sintomas respiratórios foram relatados por 58% das crianças e gastrintestinais por 34%. Três crianças apresentavam-se assintomáticas, 81 (70%) com sintomas de vias aéreas superiores, 15 (13%) com quadro de pneumonia leve e 16 (14%) com pneumonia grave. As crianças hospitalizadas eram mais jovens do que as não hospitalizadas (7 meses *vs*. 36 meses). Nas hospitalizadas, observamos maior frequência de irritabilidade, dispneia, sonolência, desconforto respiratório, baixa saturação de oxigênio e hepatomegalia. Radiografia de tórax foi realizada por 69 crianças com 45% de exames alterados. Nenhuma criança necessitou de ventilação mecânica e não houve óbitos.

**Conclusões:**

Observamos que crianças e adolescentes acometidos pela Covid-19 apresentaram, em sua maioria, quadros leves e limitados a sintomas de via aérea superior. A gravidade do quadro clínico da Covid-19 foi maior entre as crianças de menor idade que tinham com maior frequência sintomas gastrintestinais e pulmonares.

## INTRODUCTION

COVID-19 is the term used to define the disease caused by the new coronavirus, the SARS-CoV-2. This virus originated in the city of Wuhan, Hubei Province, China, in late 2019 and quickly spread throughout Europe, the United States of America, and other Latin American countries.^1^ In March 2020, the World Health Organization (WHO) declared a pandemic.

On February 26, 2020, the first case of COVID-19 was diagnosed in Brazil, in the city of São Paulo (state of São Paulo). As of June 30, 2020, the date on which the collection of cases for this study was completed, Brazil had 1,402,041 confirmed cases of COVID-19, according to the Brazilian Ministry of Health, with 59,595 deaths.^2^ On that date, the state of São Paulo recorded 281,380 cases, of which 15,322 were in the pediatric age group (from 0 to <18 years), 5.5% of the total cases in the state.^3^

In adults, COVID-19 is characterized by respiratory system involvement, which, in most severe cases, progresses with pneumonia and severe acute respiratory syndrome.^4^ ^,^ ^5^ For reasons that have not yet been fully known, the infection of COVID-19 among children and adolescents is milder, and lethality is much lower.^1^ ^,^ ^6^ ^,^ ^7^

Studies describing the manifestations of COVID-19 in children are still scarce, and the number of reported cases is often small.^7^ ^,^ ^8^ The objectives of this study were to describe the clinical manifestations and severity of children and adolescents affected by COVID-19 treated or hospitalized in a single private hospital in the city of São Paulo and also to compare such manifestations between younger and older children.

## METHOD

This is a cross-sectional, retrospective, and observational study. Patients seen in the emergency room and hospitalized in the first-aid room and in the Intensive Care Unit (ICU) of Sabará Hospital Infantil were analyzed. This is a private and exclusively pediatric hospital, part of the supplementary health system and located in the city of São Paulo, state of São Paulo, Brazil. The institution comprises 135 beds and provides about 140,000 medical services per year.

We selected all medical services provided to children and adolescents of both sexes, aged 0 to <18 years, between March 1 and June 30, 2020 and who had a diagnosis of COVID-19 (ICD: B34.2).

The medical forms and records were reviewed by a pediatric physician, who collected data on the reported symptoms, clinical signs found on physical examination, performed tests, prescribed medications, epidemiological history, and progress.

Confirmed cases were deemed those with clinical diagnosis of COVID-19 and positive RT-qPCR test for SARS-CoV-2. The severity of the cases was classified according to the Chinese Consensus:

- Asymptomatic: children without symptoms.
- Upper Respiratory Tract Infection (URTI): children with respiratory symptoms, but no changes in chest imaging and no sepsis.
- Mild pneumonia: children with respiratory symptoms and images suggestive of viral pneumonia, but no criteria for severe pneumonia.
- Severe pneumonia: children with tachypnea, low oxygen saturation (≤93%), dyspnea, altered level of consciousness, dehydration, or tomographic imaging with bilateral or multifocal pulmonary infiltrates.
- Critical: children who required ICU treatment with mechanical ventilation, shock, or multiple organ failure.^9^

The study was submitted and approved by the Research Ethics Committee of the institution (Certificate of Presentation for Ethical Consideration [CAAE] 31155920.3.0000.5567).

Continuous variables were described by median and interquartile range (IQR) and categorical variables, by percentage. Frequency comparisons were made by Chi-square and Fisher’s exact tests, whereas the comparison of continuous variables was performed by the Mann-Whitney U test. Significance was considered when p<0.05, and the program used for statistical analysis was the *Statistical Package for the Social Sciences* (SPSS), version 22.

## RESULTS

Of the 126 children and adolescents diagnosed with COVID-19, 11 were excluded from the study, two due to diagnosis established only by serology and nine due to lack of clinical data in the medical records. Among the 115 children included, a predominance of boys (57,4%) was verified, and the median age was two years (IQR=11 months-8 years). A total of 22 (19.1%) children were hospitalized and, of these, 12 (10.4%) required ICU care. Hospitalizations lasted from two to 11 days (median=4 days).

Some comorbidity was reported in 30 (26.1%) cases, with a predominance of asthma (13%), other allergies (7%), and prematurity (4.3%). Inhaled corticosteroid (7.8%) and leukotriene receptor antagonist (6.1%) were the most frequent types of continuous medication. In 86 (74.7%) cases, contacts’ tests classified as positive or suspect for COVID-19 were reported, with a wide predominance of parents (65 cases; 75.6%).

Fever, cough, and nasal discharge were the most frequently reported symptoms, as demonstrated in Table 1. Respiratory symptoms were reported by 67 (58.3%) children, and gastrointestinal symptoms, by 39 (33.9%). Five children presented skin lesions, four with exanthem and one with hives.

**Table 1:** Main symptoms and signs exhibited by children and adolescents evaluated and classified according to the need or not for hospitalization.

Characteristic	Total n=115 (%)	Hospitalized n=22 (%)	Non-hospitalized n=93 (%)	p-value
Fever	68.7	77.3	66.7	0.24
Nasal discharge	57.4	54.5	58.1	0.47
Cough	51.3	63.6	71.4	0.15
Inappetence	21.7	36.4	18.3	0.06
Headache	20.0	4.5	23.7	0.03
Oropharynx hyperemia	20.0	9.1	22.6	0.13
Nausea/vomiting	17.4	27.3	15.1	0.15
Dyspnea	13.9	45.4	6.5	0.001
Respiratory distress	13.9	40.9	7.5	0.001
Diarrhea	13.0	22.7	10.8	0.13
Irritability	10.4	31.8	5.4	0.02
Sore throat	9.6	4.5	10.8	0.34
Myalgia	9.6	4.5	10.8	0.36
Abdominal pain	8.7	4.5	9.7	0.39
Drowsiness	6.1	18.2	3.2	0.02
Anosmia	5.2	4.5	5.4	0.68
Skin lesions	4.3	9.1	3.2	0.24
SpO_2_<93%	4.3	18.2	1.1	0.004
Enlarged lymph nodes	3.5	9.1	2.2	0.17
Ageusia	2.6	0.0	3.2	0.53
Hepatomegaly	2.6	13.6	0.0	0.006
Wheezing	2.6	9.1	1.1	0.09

Regarding severity, three (2.6%) children were asymptomatic; 81 (70.4%) were classified as having URTI only; 15 (13%), with mild pneumonia; and 16 (14%), with severe pneumonia. No child was classified as in a critical condition. Children with URTI were significantly younger than those with moderate and severe conditions (median=36 months [12-96] *vs*. 12 months [2-36]; p=0.004).

The median age of hospitalized children was significantly lower than that of non-hospitalized children (7 months [IQR=2-12] *vs*. 36 months [12-96]; p=0.001). When comparing symptoms and signs between hospitalized and non-hospitalized children, a significantly higher frequency of irritability, dyspnea, drowsiness, respiratory distress, low oxygen saturation, and hepatomegaly was observed among hospitalized patients; and headache was verified among non-hospitalized ones.

A total of 58 (50.4%) children were under the age of three and were classified as younger children. These were hospitalized the most and required ICU care more frequently, as demonstrated in Table 2. When compared with older children, younger children had fever, cough, inappetence, nausea and/or vomiting, diarrhea, respiratory distress, dyspnea, and irritability more frequently. In addition, there was a lower frequency of abdominal pain, sore throat, myalgia, headache, and anosmia among them (Table 2).

**Table 2:** Main characteristics, symptoms, and signs exhibited by younger children (age≤2 years) and older children and adolescents (age≥3 years) evaluated.

Characteristic	≤2 years n=58 (%)	≥3 years n=57 (%)	p-value
Boys	60.3	54.4	0.32
Hospitalization	31.0	7.0	0.001
ICU	17.2	3.5	0.02
Comorbidity	20.7	31.6	0.23
Fever	81.0	56.1	0.004
Nasal discharge	72.4	42.1	0.001
Cough	63.8	38.6	0.006
Inappetence	36.2	7.0	0.001
Nausea/vomiting	25.9	8.8	0.02
Diarrhea	24.1	1.8	0.001
Respiratory distress	20.7	7.0	0.03
Dyspnea	19.0	8.8	0.09
Irritability	17.2	3.5	0.02
Oropharynx hyperemia	13.8	26.3	0.07
Drowsiness	10.3	1.8	0.06
Skin lesions	6.9	1.8	0.19
Hepatomegaly	5.2	0.0	0.13
SpO_2_<93%	5.2	3.5	0.52
Enlarged lymph nodes	3.4	3.5	0.68
Abdominal pain	3.4	14.0	0.04
Wheezing	3.4	1.8	0.51
Sore throat	1.7	17.5	0.004
Myalgia	1.7	17.5	0.004
Headache	1.7	38.6	0.001
Anosmia	0.0	10.5	0.01
Ageusia	0.0	5.3	0.12

ICU: Intensive Care Unit.

No difference was verified in clinical manifestations between boys and girls, nor in the frequency of hospitalization and ICU care.

A total of 69 children underwent chest radiograph, and 31 (44.9%) presented altered results, with predominance of images compatible with perihilar peribronchovascular thickening (28 cases) and localized pulmonary opacities (six cases). Of the six patients who underwent chest tomography, four had altered results, all with ground-glass opacities.

A total of 24 patients were treated with antibiotics, 15 with monotherapy and nine with the combination of two (six cases) or three (three cases) different antibiotics. Azithromycin (n=12) was the most widely used antibiotic, followed by ceftriaxone (n=11), amoxicillin clavulanate (n=8), cefuroxime (n=2), clarithromycin (n=2), and levofloxacin (n=1). Oxygen therapy was required for seven of the 22 hospitalized children. No child required mechanical ventilation and there were no deaths.

A total of 19 children underwent rapid influenza diagnostic tests (Influenza A and B), all with negative results. Of the 11 children who participated in a respiratory virus research panel, only one had a positive result (respiratory syncytial virus - RSV) - a newborn hospitalized in the ICU.

## DISCUSSION

The results of this study corroborate previous findings stating that children affected by COVID-19 present milder clinical conditions and lower mortality than adults.^1^ ^,^ ^4^ ^,^ ^7^ Approximately 70% of children affected by COVID-19 reported a condition of URTI, without involvement of the lower airways. However, it is worth noting that, even with a milder manifestation, 27% of the cases had pneumonia and 19% were hospitalized, 10% in the ICU, with indication of intensive care on the part of the team that cared for the children. The studied group of children was formed by those who sought care in the emergency room or were hospitalized, thus reflecting the clinical manifestations of patients most severely affected. It is reasonable to speculate that a large group of asymptomatic or oligosymptomatic children have not even sought medical care.

Fever, nasal discharge, and cough were the most common symptoms and the only ones that affected more than half of children. When disregarding general symptoms, respiratory symptoms were the most frequent (58.3%), followed by gastrointestinal manifestations (33.9%). This pattern of symptoms is similar to that described by the pediatric largest case series, mainly from China and Europe, and by systematic reviews.^7^ ^,^ ^8^ ^,^ ^10^ ^,^ ^11^ ^,^ ^12^

The severity of the clinical condition was clearly higher among younger children, aging up to 3 years, with a frequency of hospitalization four times higher than that of older children, and who required ICU care five times more (Table 2). This finding corroborates the results found in the pediatric largest case series published to date. In the study on over 2,000 Chinese children infected with SARS-CoV-2, the proportion of severe cases progressively decreases with increasing age, ranging from 10% in children under one year of age to 3-4% in adolescents.^10^

Among younger children, a higher frequency of some general symptoms was observed, such as inappetence and irritability, but also respiratory manifestations, such as cough and respiratory distress, and gastrointestinal manifestations. The frequency of diarrhea was markedly higher among children under three years of age (24.1 *vs.* 1.8%). On the other hand, older children had a higher frequency of painful symptoms (abdominal pain, sore throat, and headache) and of changes in smell and taste, common symptoms among adults. It is worth highlighting that this comparison should be carefully interpreted, considering that painful symptoms and altered smell and taste are hardly reported by younger children or perceived by the parents.

Similar to other pediatric studies,^7^ ^,^ ^10^ ^,^ ^11^ ^,^ ^12^ a higher frequency of involvement of boys was also observed, but without differences in clinical severity and manifestations. In approximately 75% of the cases, some family member had a positive or suspect diagnosis, reinforcing the reports according to which children were mostly contaminated at home.^11^ ^,^ ^12^

Asthma and other allergies were the most common comorbidities between children in this study. As reported in a study on 182 children hospitalized due to COVID-19 in China,^11^ no greater severity of cases was found among asthmatic and allergic children (data not shown). Respiratory infections are the main triggers of asthma exacerbations in children, and asthmatics are usually more severely affected by these infections and develop more complications. In the present study, only two of the 15 asthmatic patients presented wheezing after being diagnosed with COVID-19. This atypical behavior of SARS-CoV-2 infection is not yet fully known, but there is evidence that the lower expression of the virus cell receptor (ACE2) in airway cells of children and adults with asthma and/or respiratory allergy contributes to the lower severity of the clinical conditions.^13^

Several studies have reported the infrequent event of co-infection with SARS-CoV-2 and other viruses. Although it is not the purpose of this study and there was no systematic research for other respiratory viruses, one of the 11 cases in the respiratory virus research panel was positive for RSV. None of the rapid influenza diagnostic tests were positive. In a Chinese study, co-infection was found in 22% of cases, with predominance of *Mycoplasma pneumoniae* (19%) and cytomegalovirus (3%).^11^ In a European multicenter study, co-infection was found in 5% of pediatric cases and consisted in a risk factor for requiring ICU treatment,^12^ as observed in the only case of the present study.

In this study, chest radiographs showed altered images in about 45% of the cases in which this examination was performed. The observed frequency of alterations was lower than that observed in adults^4^ and, overall, the outcomes were less severe. In a compilation of 582 cases of children from Europe, 34% out of the total underwent chest radiograph; of these, 47% had signs of pneumonia,^12^ a value very close to that found in this study. In the present study, ground-glass opacities were the most frequent alterations found in the chest tomography examinations, similar to what has been observed in adults.^4^ Nevertheless, the small number of performed tests (n=6) does not allow for more solid conclusions.

More recently, cases of children with mucocutaneous lesions similar to those found in Kawasaki disease and inflammatory involvement of multiple organs and/or systems associated with SARS-CoV-2 infection have been reported.^14^ These severe clinical conditions with frequent involvement of the cardiovascular system have been called multisystem inflammatory syndrome in children. In this study, only one child presented a condition compatible with this syndrome.

The study has some limitations. Data were retrospectively collected from medical forms and records, and there may be inaccuracies and omissions in the data. Data on the clinical progress of non-hospitalized children may be incomplete in cases in which medical care was sought in another hospital. Despite these limitations, this study is, to the best of the authors’ knowledge, one of the pediatric largest case series outside China. Knowing particularities of clinical manifestations in children may contribute to the diagnosis and management of COVID-19 cases in children and adolescents.

In conclusion, it is noteworthy that most children and adolescents affected by COVID-19 who reside in São Paulo had mild clinical conditions that were limited to upper airway symptoms. Pneumonia was observed in 27% of the cases and there were no deaths. Hospitalization was required in 19% of the cases and, among hospitalized patients, there was a higher frequency of respiratory symptoms. The COVID-19 clinical condition was more severe among young children, who exhibited general, respiratory, and gastrointestinal symptoms more frequently.
